# Do Cuticular Gaps Make It Possible to Study the Composition of the Cell Walls in the Glands of *Drosophyllum lusitanicum*?

**DOI:** 10.3390/ijms25021320

**Published:** 2024-01-21

**Authors:** Bartosz J. Płachno, Małgorzata Kapusta, Piotr Stolarczyk, Piotr Świątek

**Affiliations:** 1 Department of Plant Cytology and Embryology, Institute of Botany, Faculty of Biology, Jagiellonian University in Kraków, 9 Gronostajowa St., 30-387 Kraków, Poland; 2 Bioimaging Laboratory, Faculty of Biology, University of Gdańsk, 59 Wita Stwosza St., 80-308 Gdańsk, Poland; malgorzata.kapusta@ug.edu.pl; 3 Department of Botany, Physiology and Plant Protection, Faculty of Biotechnology and Horticulture, University of Agriculture in Kraków, 29 Listopada 54 Ave., 31-425 Kraków, Poland; piotr.stolarczyk@urk.edu.pl; 4 Institute of Biology, Biotechnology and Environmental Protection, Faculty of Natural Sciences, University of Silesia in Katowice, 9 Bankowa St., 40-007 Katowice, Poland; piotr.swiatek@us.edu.pl

**Keywords:** arabinogalactan proteins, carbohydrate epitopes, carnivorous plants, cell wall, cuticle, cuticular gaps, Drosophyllaceae, mucilage glands, transfer cells, scanning transmission electron microscopy

## Abstract

Carnivorous plants can survive in poor habitats because they have the ability to attract, capture, and digest prey and absorb animal nutrients using modified organs that are equipped with glands. These glands have terminal cells with permeable cuticles. Cuticular discontinuities allow both secretion and endocytosis. In *Drosophyllum lusitanicum*, these emergences have glandular cells with cuticular discontinuities in the form of cuticular gaps. In this study, we determined whether these specific cuticular discontinuities were permeable enough to antibodies to show the occurrence of the cell wall polymers in the glands. Scanning transmission electron microscopy was used to show the structure of the cuticle. Fluorescence microscopy revealed the localization of the carbohydrate epitopes that are associated with the major cell wall polysaccharides and glycoproteins. We showed that *Drosophyllum* leaf epidermal cells have a continuous and well-developed cuticle, which helps the plant inhibit water loss and live in a dry environment. The cuticular gaps only partially allow us to study the composition of cell walls in the glands of *Drosophyllum*. We recoded arabinogalactan proteins, some homogalacturonans, and hemicelluloses. However, antibody penetration was only limited to the cell wall surface. The localization of the wall components in the cell wall ingrowths was missing. The use of enzymatic digestion improves the labeling of hemicelluloses in *Drosophyllum* glands.

## 1. Introduction

Carnivorous plants have the ability to attract, capture, and digest prey using modified organs—traps—and then absorb the nutrients that are obtained from the bodies of the victims [1,2,3]. Typical carnivorous plants occur in swamps, bog habitats, or humid tropical rainforests [4,5,6,7,8,9]. There are also completely aquatic carnivorous plant species [10,11,12,13]. In these habitats, there is a wide diversity of carnivorous plant species. This is because carnivory is most likely to evolve and be favored ecologically (optimal investment in carnivory) in these sunny, nutrient-poor habitats that are rich in water [14,15]. However, in contrast to most carnivorous plants, *Drosophyllum lusitanicum* (L.) Link inhabits heathlands and ruderal sites with both very tough climatic and pedological conditions. In these places, *Drosophyllum* is exposed to an extreme climate with very high air and soil temperatures and very low air humidity throughout the day [16,17,18]. According to Adamec [19], *Drosophyllum* tissues do not store water. *Drosophyllum* has a xeromorphic root system [19,20,21] that is not able to supply the plant with sufficient water to cover the transpiration rates when the plant is exposed to an extreme temperature and the soil is dry [19]. The key to their survival are the stalked, glandular emergences (glands) that occur on the *Drosophyllum* leaves. These produce a polysaccharide mucilage that is hygroscopic [4,22,23], and water then condenses from the oceanic fog as dew onto these mucilage droplets. Adlassnig et al. [16] and Adamec [19] proposed that this water can then be absorbed by the glands and transported to the leaves; thus, plants can survive harsh conditions. This mucilage is also used to trap prey [24,25] and contains volatile organic compounds, so it has a stake in attracting victims [26]. Experimentally, it was shown that *Drosophyllum* has been found to take up nutrients (N, P, K, and Mg) from insects [27].

Due to their peripheral location, the gland cells of carnivorous plants are easily accessible to fixatives, which is extremely important for keeping the cell structure intact. They are also rich in cytoplasm and display a wide range of cellular activities, and therefore all of these characteristics make the cells of the carnivorous plant glands an interesting research model [28]. Rottloff et al. [29,30] showed the carnivorous plant glands could be easily isolated and directly used for further gene expression analysis using PCR techniques after preparing the RNA. Carnivorous plants have glands with permeable cuticles because of the occurrence of cuticular discontinuities that allow secretion and endocytosis to occur [4]. In some carnivorous plants, endocytosis has been confirmed as one way that they absorb the nutrients from the bodies of captured prey [31,32,33]. There are several types of these discontinuities [4,34,35,36,37]. For example, in the glandular cells of the tentacles of *Drosera*, there are cuticular pores (about 30 nm in diameter), which can be visualized using SEM [38,39]. Similar pores were also observed in the glands of *Genlisea* [40,41] and in the epidermal cells of *Roridula* [42]. In the mucilage glands of *Drosophyllum lusitanicum*, cuticular discontinuities were found by Schnepf [43], who later described them as cutin-free wall regions that are invisible in the SEM. The term ‘cuticular gaps’ was proposed for such discontinuities [36,37]. Cuticle discontinuities in carnivorous plant glands are easily detected using vital dyes, e.g., an aqueous solution of neutral red or methylene blue [24,31,36,37,40]. Moreover, these cuticle discontinuities in carnivorous plant glands allow intact cells to be penetrated by dyes such as DiOC_6_ and styryl dye FM4-64, thereby making it possible to visualize the organelles and membranes, as was shown by Płachno et al. [41], Lichtscheidl et al. [44], and Li et al. [45]. Thus, these cuticle discontinuities make the cells of the carnivorous plant glands an excellent research model.

Cell wall polymers play an important role in the functionality of plant cell walls. For example, esterified homogalacturonans are involved in the porosity, elasticity, expansibility, and hydration of the cell wall [46,47,48,49,50]. De-esterified homogalacturonans increase the rigidity and resistance to mechanical stress of the cell walls [47,50,51]. Glactan also plays a role in cell wall rigidity [50,52]. Hemicelluloses contribute to strengthening the cell wall by interacting with cellulose [53]. Arabinogalactans have many important functions in plant cells that range from participating in the formation of wall outgrowths to playing the role of signal molecules [54,55,56,57,58].

Recently, using sectioned material, we showed the occurrence of the major cell wall polysaccharides and glycoproteins in the mucilage glands of *Drosophyllum lusitanicum* [59]. In this study, we wanted to assess whole-mount immunolabeled glands using the same species. We wanted to determine whether the specific discontinuous cuticle of the secretory cells of glands is permeable enough to antibodies. Knowing the occurrence of wall components in the sliced material where the walls are accessible to antibodies, we compared them with the whole-mount immunolabeling method, which was mainly used for root hairs and pollen tubes [60,61,62,63,64]. Recently, we used this method in another carnivorous plant in order to localize the cell wall components in the trichomes from *Utricularia dichotoma* traps [65]; however, we had only partial success as the labeling was limited to the glandular cell fragments in which the cuticle was discontinuous.

## 2. Results

### 2.1. Cuticle and Cuticular Discontinuities

**Figure 1:**
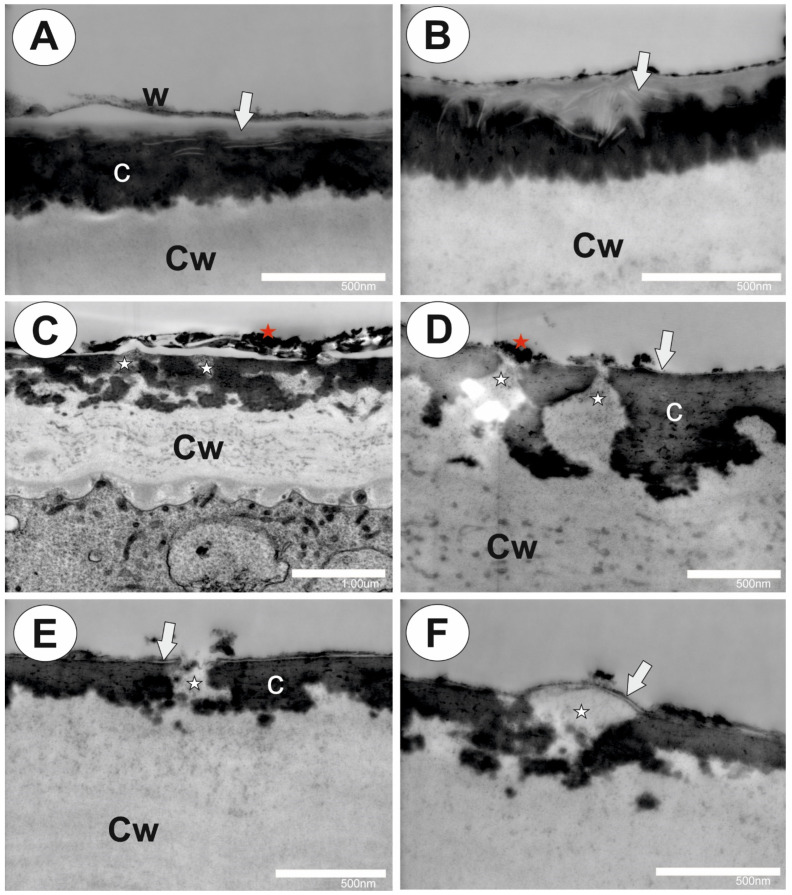
The structure of the cuticle and cuticular discontinuities of the epidermal cells and glands of *Drosophyllum lusitanicum*. (**A**,**B**) STEM of a mature *Drosophyllum lusitanicum* epidermal cell showing the cell wall and cuticle, bars 500 nm and 500 nm. (**C**) STEM of the outer glandular cell of a stalked mucilage gland showing the cuticular gaps with wall elements that extend to the cell surface, bar 1 µm. (**D**) STEM of the outer glandular cell of the mucilage gland showing cuticular gaps (white star) with wall elements that extend to the cell surface, bars 500 nm and 500 nm. (**E**) STEM of the outer glandular cell of the sessile digestive gland showing the cuticular gap (white star); note the cuticularized wall layer (arrow); bar 500 nm. (**F**) STEM of the outer glandular cell of the sessile digestive gland showing a large cuticular gap (white star) covered by a cuticularized wall layer (arrow), bar 500 nm. Abbreviations: w—wax; Cw—cell wall; c—cutinized wall; arrow—cuticularized wall layer; red star—wax or other external detritus; white star—cuticular gap.

**Figure 2:**
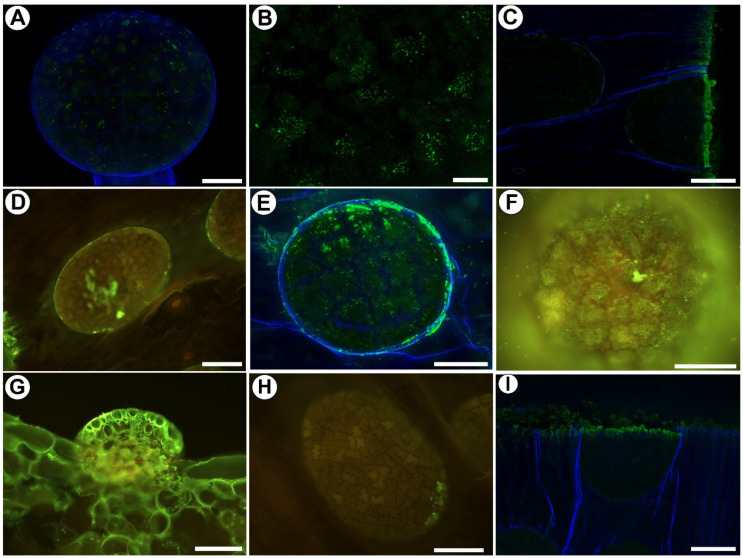
The arabinogalactan proteins that were detected in the glands of *Drosophyllum lusitanicum*. Green fluorescence is a sign of antibodies. (**A**) Arabinogalactan proteins (labeled with JIM8, green fluorescence) were detected in the mucilage gland, bar 50 µm. (**B**) Arabinogalactan proteins (labeled with JIM8) were detected in the outer glandular cell of the mucilage gland, bar 10 µm. (**C**) JIM8 fluorescence signals in the cell walls of the sectioned cells of the leaf, bar 50 µm. (**D**) JIM8 fluorescence signals in the debris on the digestive gland surface, bar 100 µm. (**E**) Arabinogalactan proteins (labeled with JIM13, green fluorescence) were detected on the digestive gland surface, bar 50 µm. (**F**) Arabinogalactan proteins (labeled with JIM13) were detected on the mucilage gland surface, bar 100 µm. (**G**) JIM13 fluorescence signals in the cell walls of the sectioned cells of the leaf and digestive gland, bar 100 µm. (**H**) JIM14 fluorescence signals in the debris on the digestive gland surface, bar 100 µm. (**I**) JIM14 fluorescence signals in the cell walls of the sectioned cells of the leaf, bar 50 µm.

The epidermal cells of mature leaves have a well-developed cuticle with a continuous cuticularized wall layer and a thick cutinized wall. The cuticularized wall layer contains electron-translucent lamellae, which are parallel to the surface. The cutinized wall also contained some electron-translucent lamella. There was also cuticular wax (Figure 1A,B). In the mucilage gland head cells, there is a well-developed cutinized wall, however, with some parts that are cutin-free. These regions without cutin (cuticular gaps) were connected to the cell surface (Figure 1C,D). The cuticular gaps were up to 450 nm (130–450 nm) in diameter. The cuticular material/wax, or other external detritus were observed on the cell surface (Figure 2C). In some glands, a thin, cuticularized wall layer also occurred (Figure 2D). In the digestive gland head cells, there was a cuticle with a thin, cuticularized wall layer and a thick, cutinized wall. Cuticular gaps also occurred, and they were up to 450 nm (60–450 nm) in diameter (Figure 2E). Some cuticular gaps were covered by a cuticularized wall layer (Figure 2F).

### 2.2. Distribution of Arabinogalactan Proteins (AGPs)

We used the JIM8, JIM13, and JIM14 antibodies in order to localize the AGPs. JIM8 reacted with the fluorescence in the cell walls of the outer glandular cell of the stalked mucilage glands (Figure 2A,B). JIM8 reacted with a strong fluorescence in the cell walls of the sectioned cells of the leaf and digestive glands (Figure 2C). JIM8 yielded fluorescence signals in the debris on the cell surfaces (Figure 2D). JIM13 yielded fluorescence signals in the debris or secretions on the epidermal cell surfaces and also gland cells (Figure 2E). JIM13 reacted with a strong fluorescence in the cell walls of the sectioned cells of the leaf and digestive glands (Figure 2G). JIM14 yielded fluorescence signals in the debris on the cell surfaces (Figure 2H) and in the cell walls of the sectioned cells of the leaf (Figure 2I).

### 2.3. Distribution of Homogalacturonan

**Figure 3:**
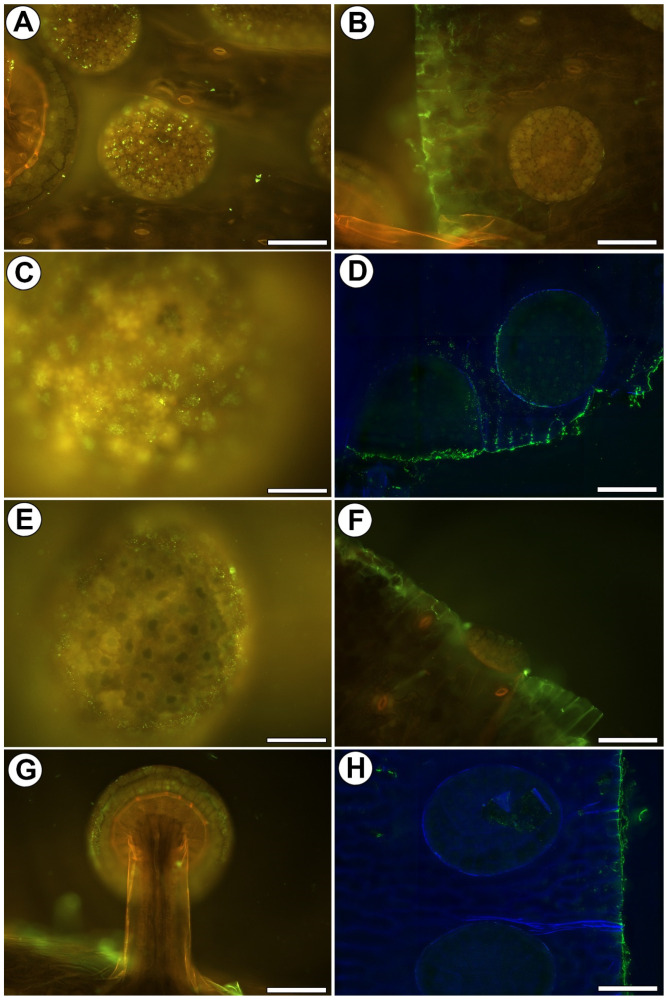
Homogalacturonan (HG) was detected in the glands of *Drosophyllum lusitanicum*. Green fluorescence is a sign of antibodies. (**A**) JIM5 green fluorescence signals in the debris on the cell surfaces, bar 100 µm. (**B**) JIM5 fluorescence signals in the cell walls of the sectioned cells of the leaf, bar 100 µm. (**C**) Homogalacturonan (labeled with LM19) was detected in the mucilage gland cells, bar 50 µm. (**D**) LM19 fluorescence signals in the cell walls of the sectioned cells of the leaf and cell surfaces, bar 100 µm. (**E**) A weak signal (spot signal) of JIM7 in the external cell walls of the outer glandular cells of mucilage gland, bar 50 µm. (**F**) Signal of JIM7 in cell walls in the sectioned cells of the leaf, bar 100 µm. (**G**) A weak signal (spot signal) of LM5 in the external cell walls of the outer glandular cells of mucilage gland, bar 100 µm. (**H**) Signal of LM5 in cell walls in places where cuticles were damaged in digestive gland and in the sectioned cells of the leaf, bar 100 µm.

Low-methyl-esterified Homogalacturonans (HG) were detected by JIM5 and LM19 antibodies. JIM5 yielded fluorescence signals in the debris on the cell surfaces (Figure 3A) and in the cell walls of the sectioned cells of the leaf (Figure 3B). The fluorescence signal that was detected by LM19 occurred in the external cell walls of the outer glandular cells of the mucilage glands (Figure 3C). It also occurred in the debris on the cell surfaces and in the cell walls of the sectioned cells of a leaf (Figure 3D).

Highly esterified HGs were detected by JIM7 antibodies. A fluorescence signal was observed in the external cell walls of the outer glandular cells of the mucilage glands (Figure 3E) and in the cell walls of the sectioned cells of the leaf (Figure 3F).

The pectic polysaccharide (1–4)-β-D-galactan was detected by LM5. A weak signal (spot signal) occurred in the external cell walls of the outer glandular cells of both gland types (Figure 3G). In the areas where the cuticle was damaged, the signal was stronger (Figure 3H).

### 2.4. Distribution of Hemicellulose

**Figure 4:**
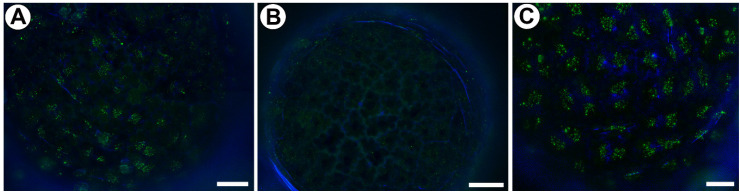
Xyloglucan was detected in the glands of *Drosophyllum lusitanicum*. Green fluorescence is a sign of antibodies. (**A**) Xyloglucan (labeled with LM15) detected in the mucilage gland cells after they had been pre-treated with pectate lyase, bar 20 µm. (**B**) Xyloglucan (labeled with LM25) detected in the mucilage gland cells bar 20 µm. (**C**) Xyloglucan (labeled with LM25) detected in the mucilage gland cells after they had been pre-treated with pectate lyase, bar 20 µm.

Xyloglucan was detected by LM15 and LM25 antibodies. The LM15 antibody gave a fluorescence signal in the cell walls of the sectioned cells of the leaf and gland. When the sections were pre-treated with pectate lyase (during which the pectins were removed), the LM15 antibody gave a fluorescence signal in the external cell walls of the outer glandular cells of the mucilage glands (Figure 4A). LM25 gave a weak fluorescence signal from xyloglucan in the walls of the outer glandular cells of the mucilage glands (Figure 4B). When the sections were pre-treated with pectate lyase (during which the pectins were removed), the LM25 antibody gave a stronger fluorescence signal in the external cell walls of the outer glandular cells of the mucilage glands (Figure 4C).

## 3. Discussion

### 3.1. Cuticle Structure

Adamec [19] suggested that *Drosophyllum* leaves have a thick cuticle that efficiently prevents water losses via transpiration. In this study, we proved this because we found a thick, continuous, and well-developed cuticle (with a continuous cuticularized wall layer and a thick cutinized wall) in the epidermal cells, which acts as a barrier to excessive water loss. Similar to the observations of Joel and Juniper [36], Juniper et al. [4], and Vassilyev [66], we found cuticular gaps in the cuticle of mature *Drosophyllum* gland cells. However, we observed that some of these cuticular gaps were covered by a cuticularized wall layer, which may call into question whether these gaps are functional. Perhaps such discontinuities are still emerging, and those that are covered by a cuticularized wall layer are in a state of immaturity.

### 3.2. Pros and Cons of the Whole-Mount Immunolabeled Gland Technique

A cuticularized wall layer is probably a barrier to antibodies. Anderson [42] observed that in *Roridula*, some gaps have a plug of either cuticular wax or other external detritus. He also wondered whether such gaps would be permeable to liquid compounds. We also observed external detritus on the gland cell surface. Such material can block the access of antibodies to the cell walls. In addition, as we also showed, such material reacts with some of the antibodies, which may be due to the fact that it is partly derived from the plant’s secretions. In the trichomes from *Utricularia dichotoma* traps, Płachno and Kapusta [65] showed that the success of the labeling was connected to the permeability of the cuticle (the occurrence of discontinuities). In the case of *Drosophyllum*, some of the results are ambiguous, and it is sometimes difficult to distinguish whether the signal came from the peripheral part of the wall or from the secretion or the material on the cell surface. Therefore, it is interesting to compare the occurrence of the cell wall components in the cells of the outer mucilage glands, which was our method, with the traditional method used by Płachno et al. [59] (Table 1). As for the AGPs, their detection was possible using both methods. However, in the sliced material, the AGPs were also found in the cell wall ingrowths. We know from our previous analyses of the glands of carnivorous plants that cell wall growths contain arabinogalactans. This has been found in the glands of *Dionaea muscipula* [67,68] and *Aldrovanda vesiculosa* [69,70]. AGPs have also been reported in cell wall ingrowths in various plant species, both in lower plants and angiosperms [71,72,73,74,75]. In the whole-mount immunolabeled glands, there was a lack of detection of the AGPs in the deeper parts of the cell walls; thus, the detection of the AGPs in the cell wall ingrowths is ineffective. Therefore, we do not recommend this technique for the analysis of cell wall ingrowth components.

As for the occurrence of homogalacturonans, the results from both methods were similar in peripheral parts of outer cell walls, and the only difference was that in the whole-mount immunolabeled mucilage glands, signals of LM19 and JIM7 antibodies were detected. However, it should be remembered that we analyzed the surface signals, and therefore, secretion contamination cannot be ruled out. We found that using enzymatic digestion improved the labeling of hemicelluloses in the *Drosophyllum* glands. Pectins mask the presence of the hemicelluloses; thus, the digestion of pectin makes the antibodies available to the hemicelluloses [76].

We think that to better understand both the cell wall polymers and cuticle structure in *Drosophyllum* glands, immunogold techniques are required.

## 4. Materials and Methods

### 4.1. Plant Material

*Drosophyllum lusitanicum* (L.) Link plants were grown in the greenhouses of the Botanical Garden of the Jagiellonian University. The plants were kept under high sunlight exposure.

### 4.2. Histological and Immunochemical Analysis

Leaf fragments (from three plants) were fixed in 8% (*w*/*v*) paraformaldehyde (PFA, Sigma-Aldrich, Sigma-Aldrich Sp. z o.o., Poznań, Poland) mixed with 0.25% (*v*/*v*) glutaraldehyde (GA, Sigma-Aldrich, Sigma-Aldrich Sp. z o.o., Poznań, Poland) in a PIPES buffer overnight at 4 °C. The PIPES buffer contained 50 mM PIPES (piperazine-N,N′-bis [2-ethanesulfonic acid], Sigma-Aldrich, Sigma-Aldrich Sp. z o.o., Poznań, Poland), 10 mM EGTA (ethylene gly-col-bis[β-aminoethyl ether]N,N,N′,N′-tetraacetic acid, Sigma-Aldrich Sp. z o.o., Poznań, Poland), and 1 mM MgCl_2_ (Sigma-Aldrich, Sigma-Aldrich Sp. z o.o., Poznań, Poland), pH 6.8. The plant material was washed in a PBS buffer and later blocked with 1% bovine serum albumin (BSA, Sigma-Aldrich) in a PBS buffer and incubated with the following primary antibodies (Table 2) [47,76,77,78,79,80,81,82]—anti-AGP: JIM8, JIM13; JIM14; anti-pectin: JIM5, JIM7, LM19, LM5, and anti-hemicelluloses: LM25, LM15 overnight at 4 °C. All of the primary antibodies were used in a 1:20 dilution. They were purchased from Plant Probes, Leeds, UK, and the goat anti-rat secondary antibody conjugated with FITC was purchased from Abcam (Abcam plc, Cambridge, UK). The samples were then cover-slipped using a Mowiol mounting medium: a mixture of Mowiol^®^ 4-88 (Sigma-Aldrich, Sigma-Aldrich Sp. z o.o., Poznań, Poland) and glycerol for fluorescence microscopy (Merck, Poland) with the addition of 2.5% DABCO (The Carl Roth GmbH + Co. KG, Germany). The material was viewed using a Nikon Eclipse E800 microscope (Precoptic, Warsaw, Poland) and a Leica DM6000 B (KAWA.SKA Sp. z o.o., Piaseczno, Poland, and Leica Microsystems GmbH, Wetzlar, Germany). The signal of the antibodies (green fluorescence) was visualized using a Nikon B-2A filter (Longpass Emission; excitation 450–490 nm, DM. 500 nm, emission 515 nm cut-on) and a Leica GFP filter emission 525/50. The autofluorescence of cell walls and cutin was visualized with a Nikon B-2A filter (yellow fluorescence) and Leica DAPI filter emission 460/50 (blue fluorescence). Part of the photos were acquired as Z stacks and deconvolved using 5 iterations of a 3D nonblind algorithm (AutoQuant™, Media Cybernetics Inc., Rockville, Maryland, USA). In order to maximize the spatial resolution, images are presented as the maximum projections. At least two different replications were performed for each of the analyzed traps, and about five to ten glands were analyzed for each antibody that was used. Negative controls were created by omitting the primary antibody step, which caused no fluorescence signal in any of the control frames for any of the stained slides (Figure S1). To remove the HG from the cell walls, the material was pretreated with 0.1 M sodium carbonate pH = 11.4 for 2 h at room temperature. This was followed by digestion with a pectate lyase 10 A (Nzytech) at 10 μg/mL for 2 h at room temperature in 50 mM N-cyclohexyl-3-aminopropane sulfonic acid (CAPS), to which 2 mM of a CaCl_2_ buffer at pH 10 was added [76], and then incubation with the LM15 and LM25 antibodies as described above.

### 4.3. Light Microscopy (LM)

The cuticle was stained using Auramine O (Sigma-Aldrich Sp. z o.o., Poznań, Poland), and later, the traps were examined using a Leica DM6000 B (Leica Microsystems GmbH, Wetzlar, Germany) and also a Nikon Eclipse E400 light microscope (Nikon, Tokyo, Japan) with a UV-2A filter (Ex. 330–380 nm, DM. 400 nm, Em. 420-α nm).

### 4.4. Scanning Transmission Electron Microscopy

The glands were also examined using electron microscopy, as follows: Fragments of the traps were fixed in a mixture of 2.5% glutaraldehyde with 2.5% formaldehyde in a 0.05 M cacodylate buffer (Sigma-Aldrich, Sigma-Aldrich Sp. z o.o., Poznań, Poland; pH 7.2) overnight or for several days, and later, the material was processed as in Płachno et al. [83]. The material was dehydrated with acetone and embedded in an Epoxy Embedding Medium Kit (Fluka). Ultrathin sections were cut on a Leica ultracut UCT ultramicrotome. The sections were examined using a Hitachi UHR FE-SEM SU 8010 microscope, which is housed at the University of Silesia in Katowice.

## 5. Conclusions

Here we show that *Drosophyllum* leaf epidermal cells have a continuous and well-developed cuticle, which helps the plant inhibit water loss and live in a dry environment. The cuticular gaps only partially allow us to study the composition of cell walls in the glands of *Drosophyllum*. We recorded arabinogalactan proteins, some homogalacturonans, and hemicelluloses; however, antibody penetration was only on the cell wall surface. The location of the wall components in the cell wall ingrowths was missing; thus, we do not recommend the whole-mount immunolabelling organ technique for the analysis of cell wall ingrowth components. The use of enzymatic digestion improves the labeling of hemicelluloses in *Drosophyllum* glands.

## Figures and Tables

**Table 1:** Comparison of the occurrence of cell wall components in the cells of the outer mucilage glands using our method with traditional method used by Płachno et al. [59].

Antibody	Whole-Mount Immunolabeled Glands	Sectioned Glands
AGPs		
JIM8	Occurred	Occurred
JIM13	Lack of or connection with secretion	Occurred
JIM14	Lack	Weak signal
Homogalacturonan		
JIM5	Lack	Lack
LM19	Occurred	Lack or weak signal
JIM7	Occurred	Lack
LM5	Weak signal	Weak signal
	Hemicelluloses	
LM15	Occurred after being pre-treated with pectate lyase	Occurred
LM25	Occurred	Occurred

**Table 2:** List of the monoclonal antibodies that were used in the current study and the epitopes they recognize [77].

Antibody	Epitope
AGPs	
JIM8	Arabinogalactan
JIM13	Arabinogalactan/arabinogalactan protein
JIM14	Arabinogalactan/arabinogalactan protein
Homogalacturonan	
JIM5	Homogalacturonan (HG) domain of c pectic polysaccharides recognizes partially methyl-esterified epitopes of HG and can also bind to unesterified HG
JIM7	HG domain of the pectic polysaccharides recognizes partially methyl-esterified epitopes of HG but does not bind to unesterified HG
LM5	Linear tetrasaccharide in (1–4)-β-D-galactans (RGI side chain)
LM19	HG domain in pectic polysaccharides recognizes a range of HG with a preference to bind strongly to unesterified HG
	Hemicelluloses
LM15	XXXG motif of xyloglucan
LM25	XLLG, XXLG, and XXXG motifs of xyloglucan

## Data Availability

Data are contained within the article and supplementary materials.

## Supplementary Materials

The following supporting information can be downloaded at: https://www.mdpi.com/article/10.3390/ijms25021320/s1.

## Abbreviations

AGPs—arabinogalactan proteins; HG—homogalacturonans; SEM—scanning electron microscope; STEM—Scanning transmission electron microscopy; LM—light microscope.
