# Supplementary material for: Do Cuticular Gaps Make It Possible to Study the Composition of the Cell Walls in the Glands of Drosophyllum lusitanicum?
Source: Int J Mol Sci. 2024 Jan 21;25(2):1320. doi: 10.3390/ijms25021320 (PMC10816202; doi:10.3390/ijms25021320)

Figure S1

Figure S1. (**A**-**B**) Control reactions of cell wall components after immunolabeling in gland, bar 50 μm and bar 100 μm.

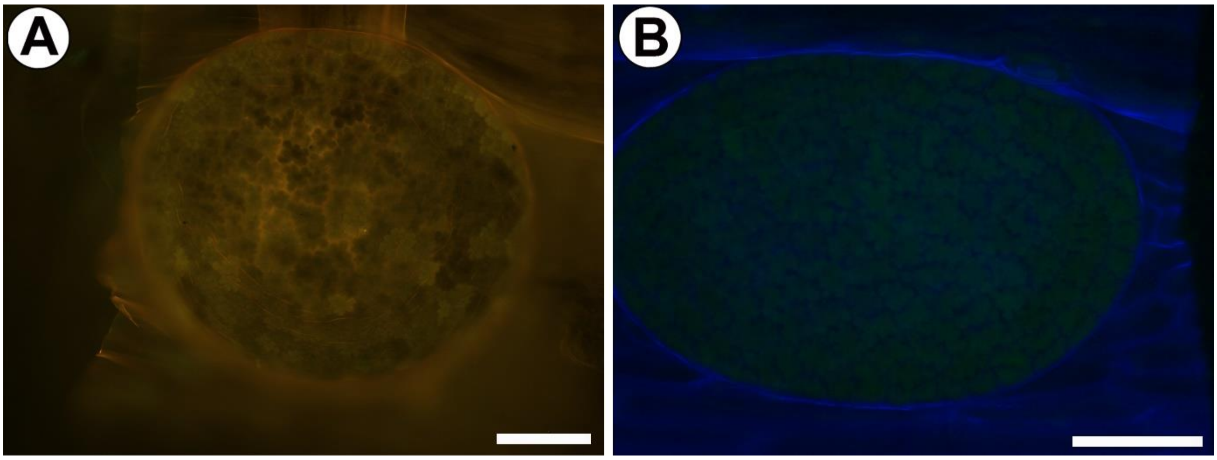

Supplement: Supplementary file 1 [file ijms-25-01320-s001.zip › ijms-2822896-supplementary.pdf]
